# Plasma Neuregulin 4 Levels Are Associated with Metabolic Syndrome in Patients Newly Diagnosed with Type 2 Diabetes Mellitus

**DOI:** 10.1155/2018/6974191

**Published:** 2018-03-12

**Authors:** Pijun Yan, Yong Xu, Qin Wan, Jian Feng, Hua Li, Jun Yang, Haihua Zhong, Zhihong Zhang

**Affiliations:** ^1^Department of Endocrinology, The Affiliated Hospital of Southwest Medical University, Luzhou, Sichuan 646000, China; ^2^Department of Cardiovascular Medicine, The Affiliated Hospital of Southwest Medical University, Luzhou, Sichuan 646000, China; ^3^Department of General Medicine, The Affiliated Hospital of Southwest Medical University, Luzhou, Sichuan 646000, China

## Abstract

Neuregulin 4 (Nrg4) has been proposed to play a role in the pathogeneses of obesity, insulin resistance, and dyslipidemia. However, information about the link between Nrg4 and metabolic syndrome (MetS) is scarce, especially in patients with newly diagnosed type 2 diabetes mellitus (nT2DM). This study aimed at investigating whether Nrg4 is associated with MetS in nT2DM patients. A total of 311 patients with nT2DM were recruited. Plasma Nrg4 concentration was determined by ELISA. Plasma Nrg4 concentration was lower in nT2DM patients with MetS than in nT2DM patients without MetS (*P* = 0.001). Nrg4 concentration showed negative correlations with most of the analyzed indicators of MetS. MetS was less prevalent among subjects in the highest quartile of plasma Nrg4 concentration than among those in the lowest quartile (*P* < 0.01). Age- and sex-adjusted plasma Nrg4 concentrations were positively correlated with concentrations of high-density lipoprotein cholesterol (HDL-C) and apolipoprotein A (both *P* < 0.05) and negatively correlated with triglyceride, high-sensitivity C-reactive protein (hs-CRP), and gamma-glutamyltransferase concentrations, neutrophil count, and white blood cell (WBC) count (all *P* < 0.05). In multivariate analysis, Nrg4 was independently associated with hs-CRP level, WBC count, and HDL-C level (*P* = 0.001 or *P* < 0.05). Multiple logistic regression analysis of MetS prediction by Nrg4 revealed an odds ratio of 0.560 (95% CI: 0.374–0.837; *P* < 0.01). Decreased plasma Nrg4 levels, which may be associated with augmented oxidative stress, inflammation, and dyslipidemia, might be involved in the development of MetS in nT2DM patients.

## 1. Introduction

Metabolic syndrome (MetS) is characterized by a cluster of metabolic disorders, including insulin resistance (IR), hyperglycemia, dyslipidemia, central obesity, and hypertension. These disorders are important risk factors of type 2 diabetes mellitus (T2DM), cardiovascular diseases, and all-cause mortality among adults and children [1, 2]. With the worldwide spread of obesity and T2DM, MetS is increasingly being seen as an important public health problem. Overall prevalence of MetS ranges from 21.9% to 49.4% in the general populations of the United States, Europe, and Asia (Thailand, Mainland China, and Hong Kong) [3]. Although IR is considered to be a key factor in the disease, the precise pathogenic mechanism of MetS is not clear. No specific or effective prevention and therapeutic strategies are available for MetS. Thus, it is imperative to identify novel biomarkers to identify MetS.

Adipose tissue is an active endocrine organ secreting a host of bioactive adipokines, including adiponectin and leptin. Adipokines modulate glucose and lipid metabolism, inflammation, and insulin sensitivity and, thus, might be involved in the pathogeneses of IR, diabetes, and MetS [4–6]. Neuregulin 4 (Nrg4) is a secreted water-soluble protein that has been found in the circulation. Nrg4 is expressed in multiple organs, with the highest expression levels in brown adipose tissue [7, 8]. A member of the epidermal growth factor (EGF) family of extracellular ligands, Nrg4 binds to and activates receptor tyrosine kinases ErbB3 and ErbB4 and acts as an autocrine, paracrine, or endocrine signal by releasing the EGF-like domain after photolytic cleavage [6–12]. Nrg4 has many biological functions, including the inhibition of apoptosis and inflammation and the promotion of neurite outgrowth [11, 13–17]. Rosell and Kaforou [17] showed that Nrg4 is upregulated in cold-induced beige/brite cells and highly expressed during brown adipocyte differentiation. Nrg4 mRNA expression was downregulated in the adipose tissues of several obese mouse models and negatively correlated with the percentage of body fat mass and liver fat content in humans [6, 8, 18]. These observations suggest that Nrg4 insufficiency may be a common feature of obesity. Furthermore, Nrg4-deficient mice fed a high-fat diet exhibited significant increases in body weight (BW), plasma triacylglyceride and fasting blood glucose levels, and worsening of IR and fatty liver. Nrg4-overexpressing mice displayed the opposite results in liver and adipose tissues [7, 8]. These results demonstrate that Nrg4 may play a crucial role in the regulation of insulin sensitivity, energy balance, and glucose and lipid metabolism.

Decreased Nrg4 levels may lead to the development of IR, T2DM, and MetS. A recent study demonstrated that Nrg4 mRNA levels in subcutaneous and visceral adipose tissues were significantly lower in patients with impaired glucose tolerance or T2DM than in normal individuals [8]. These findings suggest that Nrg4 may work as a novel adipokine protecting metabolic homeostasis. However, two recent clinical studies found that serum Nrg4 levels were significantly higher in T2DM patients than in healthy controls and were positively correlated with waist circumference (WC), fasting plasma glucose (FPG) and triglyceride (TG) concentrations, blood pressure, and IR [19, 20]. The discrepant findings might be due to differences in study design and methodology.

Recently, Dai et al. [5] indicated that patients with nonalcoholic fatty liver disease (NAFLD) had lower serum levels of Nrg4 than non-NAFLD controls. Decreased serum Nrg4 level was an independent risk factor of NAFLD, which currently is considered to be the hepatic manifestation of IR and MetS [5]. These findings strongly suggest that decreased Nrg4 may contribute to the pathogenesis of MetS. To our knowledge, only one cross-sectional study has investigated the relationship between Nrg4 and MetS in obese adults, showing decreased serum Nrg4 levels in MetS subjects with obesity [6]. No study has analyzed the association between plasma Nrg4 levels and risk of MetS in normal weight, overweight, or obese patients with T2DM. Thus, the purpose of this study was to evaluate the determinants and associations of plasma Nrg4 concentration with MetS characteristics, as defined by the Chinese Medical Association/Chinese Diabetes Society (CDS) diagnostic criteria, in newly diagnosed T2DM (nT2DM) patients.

## 2. Materials and Methods

### 2.1. Study Design and Subjects

A total of 311 patients with nT2DM were recruited to the study. Diagnosis of T2DM was made on the basis of oral glucose tolerance tests (OGTTs) and 1998 WHO diagnostic criteria. Subjects with nT2DM were not treated with hypoglycemic agents or insulin. All participants completed a standardized questionnaire regarding their medical history and lifestyle factors (smoking and alcohol) and underwent a comprehensive health examination according to standard procedures. Exclusion criteria were as follows: patients with (1) type 1 diabetes mellitus or other endocrine disorder, (2) acute complications of T2DM, gall bladder, or biliary tract disease, viral hepatitis, alcoholic liver disease, drug-induced liver disease, autoimmune hepatitis, liver and renal dysfunction, acute or chronic inflammatory disease, pregnancy, or cardiovascular or cerebral vascular disease, (3) history of use of lipid-lowering and antihypertensive drugs, and (4) total parenteral nutrition, smoking habit, or alcohol consumption. Hypertension without antihypertensive drug treatment was not an exclusion criterion. All nT2DM patients were categorized into quartiles based on their plasma Nrg4 level: quartile 1, Nrg4 < 1.97 ng/ml; quartile 2, 1.97 ng/ml ≤ Nrg4 < 2.80 ng/ml; quartile 3, 2.80 ng/ml ≤ Nrg4 ≤ 4.10 ng/ml; quartile 4, Nrg4 > 4.10 ng/ml. The study protocol followed the ethical guidelines of the 1964 Declaration of Helsinki and was approved by the human research ethics committee of the Affiliated Hospital of Southwest Medical University. Written informed consent was obtained from all patients.

### 2.2. Definition of MetS

MetS was defined by the CDS criteria [21]. For a diagnosis of MetS, participants had to meet three or more of the following criteria: (1) BW in the overweight or obese range, defined as a body mass index (BMI) ≥ 25.0 kg/m^2^; (2) hyperglycemia, defined as FPG ≥ 6.1 mmol/l and/or 2 h plasma glucose (2hPG) ≥ 7.8 mmol/l, or previously diagnosed T2DM and receiving treatment; (3) hypertension, defined as systolic blood pressure (SBP)/diastolic blood pressure (DBP) ≥ 140/90 mmHg, or previously diagnosed hypertension and receiving treatment; and (4) dyslipidemia, defined as TG ≥ 1.7 mmol/l and/or high-density lipoprotein cholesterol (HDL-C) < 0.9 mmol/l (men) or <1.0 mmol/l (women). Patients with nT2DM were considered to have MetS if they had two or more of the above factors except nT2DM.

### 2.3. Anthropometric and Biochemical Measurements

Anthropometric measurements were performed in all participants before breakfast. BW (kg), height (m), BMI, SBP, and DBP were measured by standard methods, as described previously [22]. Body fat percentage (BF%) was calculated using the following equation [23]: BF% = 1.20 × BMI − 10.8 × sex (male = 1, female = 2) + 0.23 × age (year) − 5.4.

Blood samples were collected from participants in the morning, either after an overnight fast or 2 h after beginning the 75 g OGTT. Plasma samples were collected by centrifugation at 4°C and stored at −80°C until analytical processing. FPG, 2hPG, and glycated hemoglobin A1C (HbA1c) levels were measured by the glucose oxidase method and anion-exchange HPLC, respectively. Total cholesterol (TC), TG, HDL-C, low-density lipoprotein cholesterol (LDL-C), apolipoprotein A (apoA), and apolipoprotein B (apoB) levels were analyzed enzymatically by using a 7060 full-automatic biochemical analyzer (Hitachi, Tokyo, Japan). Alanine aminotransferase (ALT), aspartate aminotransferase (AST), gamma-glutamyltransferase (GGT), and alkaline phosphatase (ALP) levels were also analyzed. White blood cell (WBC), neutrophil, and lymphocyte counts were determined by using an automated blood cell counter (Mindray BC-6800, Shenzhen, China), according to the manufacturer's instructions. The neutrophil to lymphocyte ratio (NLR) was calculated as the simple ratio between the absolute neutrophil and lymphocyte counts. High-sensitivity C-reactive protein (hs-CRP) levels were measured by latex-enhanced immunoturbidimetric assay. Fasting plasma insulin (FIns) concentrations were measured with an electrochemiluminescence immunoassay (Roche Elecsys Insulin Test, Roche Diagnostics, Mannheim, Germany). The apoB to apoA ratio (apoB/apoA) was calculated as the simple ratio between apoB and apoA. The triglyceride glucose (TyG) index was calculated as the natural logarithm of [TG (mg/dl) × FPG (mg/dl)/2] [24]. Homeostasis model assessment of insulin resistance (HOMA-IR) and homeostasis model assessment of *β*-cell insulin secretion (HOMA-IS) were calculated from FIns and FPG levels by the following formulas [4]: HOMA-IR = FIns (*μ*U/ml) × FPG (mmol/l)/22.5 and HOMA-IS = [20 × FIns (*μ*U/ml)]/[FPG (mmol/l) − 3.5].

### 2.4. Plasma Nrg4 Measurement

Plasma Nrg4 concentrations were determined with an enzyme-linked immunosorbent assay (ELISA) (Aviscera Biosciences, Santa Clara, CA), following the manufacturer's protocol. This assay has been shown to be highly sensitive to human Nrg4, with a sensitivity of 0.125–0.25 ng/ml. The linear range of the standard was 0.25–16.0 ng/ml. Intra- and inter-assay variations were both less than 10%.

### 2.5. Statistical Analysis

All analyses were performed with the Statistical Package for Social Sciences version 20.0 (SPSS, Chicago, IL). All data distributions were analyzed for normality by the Kolmogorov–Smirnov test. Data are expressed as the mean ± standard deviation (SD) for continuous variables or percentage (%) for categorical variables, unless otherwise specified. Two groups were compared by chi-square (*χ*^2^) tests for categorical variables, Student's *t*-test for normally distributed continuous variables, or Mann–Whitney *U* tests for nonparametric distributed continuous variables. More than two groups were compared by one-way analysis of variance (ANOVA) followed by the post hoc least significant difference test for normally distributed continuous variables and Kruskal–Wallis test followed by adjustment for multiple pairwise comparisons for nonparametric distributed covariates. Spearman's correlation coefficients were used to describe associations between plasma Nrg4 concentration and other variables. The partial correlation coefficient was used for sex- and age-adjusted data.

Multiple linear regression was performed to identify variables that were independently associated with plasma Nrg4 concentration. Logistic regression analysis was performed to ascertain the association of Nrg4 concentration with the presence of MetS. In logistic regression, we analyzed all potential confounding variables. Odds ratios (ORs) and 95% confidence intervals (CIs) were estimated. All reported *P* values were two-sided, and a *P* value of < 0.05 was considered statistically significant.

## 3. Results

### 3.1. Clinical and Biochemical Characteristics of nT2DM Patients with or without MetS

Table 1 summarizes the anthropometric and biochemical parameters of the 311 nT2DM patients enrolled in the cross-sectional study. Compared to nT2DM patients without MetS, nT2DM patients with MetS had higher levels of BMI, BF%, SBP, DBP, TC, TG, apoB, FPG, 2hPG, ALT, GGT, WBC count, hs-CRP, FIns, HOMA-IR, apoB/apoA, and TyG index (*P* < 0.001 or *P* < 0.01 or *P* < 0.05) and lower levels of HDL-C, apoA, and lymphocyte count (*P* < 0.001 or *P* < 0.05). Age, LDL-C, HbA1c, ALP, NLR, neutrophil count, and HOMA-IS did not significantly differ between the groups.

### 3.2. Plasma Nrg4 Levels Depending on MetS Components in nT2DM Patients

**Figure 1:**
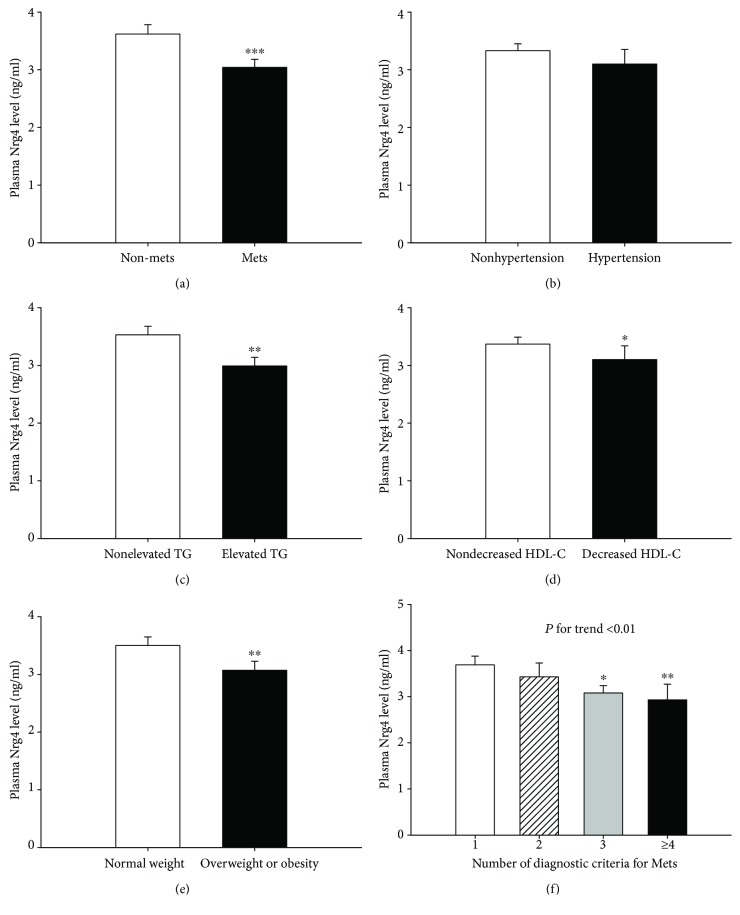
Plasma Nrg4 concentrations for nT2DM patients (a) with (*n* = 178) or without MetS (*n* = 133), (b) with (*n* = 52) or without hypertension (*n* = 259), (c) with (*n* = 141) or without elevated TG levels (*n* = 170), (d) with (*n* = 91) or without decreased HDL-C levels (*n* = 220), (e) with normal weight (*n* = 157) or with overweight or obesity (*n* = 154), (f) with one (*n* = 99), two (*n* = 34), three (*n* = 131), or four or more components of MetS (*n* = 47). ^∗^*P* < 0.05, ^∗∗^*P* < 0.01, ^∗∗∗^*P* = 0.001 versus nT2DM patients without MetS or without elevated TG levels or without decreased HDL-C levels or with normal weight or with one component of MetS.

Plasma Nrg4 levels were lower in nT2DM patients with MetS than in those without MetS (*P* = 0.001; Figure 1(a)). Furthermore, plasma Nrg4 levels were significantly lower in nT2DM patients with elevated TG levels (*P* < 0.01; Figure 1(c)), decreased HDL-C levels (*P* < 0.05; Figure 1(d)), and overweight or obese patients (*P* < 0.01; Figure 1(e)) compared to their controls. Plasma Nrg4 levels decreased in a stepwise fashion as the number of MetS components increased (*P* for trend < 0.01; Figure 1(f)). Subjects with four or five components of MetS had lower plasma Nrg4 levels than those with only one component (*P* < 0.01). However, there was no significant difference in plasma Nrg4 levels in the context of hypertension (*P* > 0.05; Figure 1(b)).

### 3.3. Clinical and Biochemical Characteristics in nT2DM Patients according to Plasma Nrg4 Quartile

Table 2 presents the clinical and biochemical characteristics distinguished by quartile of plasma Nrg4 concentration. TG, HDL-C, apoA, GGT, hs-CRP, and Nrg4 concentrations and WBC count were significantly different between patients in different plasma Nrg4 quartiles (*P* < 0.001 or *P* < 0.01 or *P* < 0.05). Compared to subjects in the lowest quartile of plasma Nrg4 concentration, patients in the highest quartile had lower levels of BMI, TG, GGT, WBC count, and hs-CRP (*P* < 0.01 or *P* < 0.05) and higher levels of plasma Nrg4, HDL-C, and apoA (*P* < 0.001 or *P* < 0.01 or *P* < 0.05). Prevalence of MetS was markedly lower in subjects in the highest plasma Nrg4 quartile than in subjects in the lowest quartile (*P* < 0.01). However, prevalence of overweight or obesity, hypertension, elevated TG levels, and decreased HDL-C levels showed no significant differences across quartiles of plasma Nrg4 concentration.

### 3.4. Linear Regression Analyses of Variables Associated with Plasma Nrg4 Levels in All nT2DM Patients

We investigated the relationship between plasma Nrg4 concentration and the other parameters. Plasma Nrg4 levels were correlated positively with HDL-C and apoA levels but negatively with TG, hs-CRP, and GGT levels and WBC and neutrophil counts (*P* ≤ 0.001 or *P* < 0.01 or *P* < 0.05). All of these correlations remained statistically significant after adjusting for sex and age (all *P* < 0.05). We performed multiple stepwise regressions to determine which variables were independently associated with plasma Nrg4 concentration. Only hs-CRP level, WBC count, and HDL-C level were independently related to plasma Nrg4 concentration (*P* = 0.001 or *P* < 0.05), with a multiple regression equation of *Y*_Nrg4_ = 3.449 − 0.194*X*_hs-CRP_ − 0.131*X*_WBC_ + 0.646*X*_HDL-C_ (Table 3).

Multivariable-adjusted ORs for the association of plasma Nrg4 concentration with increased presence of MetS are shown in Table 4. Risk of presence of MetS decreased by 15% per 1 SD increase in plasma Nrg4 levels. The OR of MetS as predicted by Nrg4 concentration in the presence of all potential confounding variables was 0.560 (95% CI: 0.374–0.837; *P* < 0.01), indicating that there was a 44% decrease in the odds of having MetS for each 1 ng/ml increase in Nrg4 levels.

## 4. Discussion

Plasma concentrations of Nrg4, a novel adipokine expressed in adipose tissues (especially brown adipose tissue), may be associated with obesity, IR, T2DM, lipid metabolism, inflammation, and atherosclerosis [6, 8, 9, 15]. When fed a high-fat diet, mice overexpressing Nrg4 specifically in the liver and adipose tissues gained significantly less weight than control mice and exhibited improvements in glucose tolerance, lipid metabolism, and insulin sensitivity, and decreased hepatic steatosis [8, 18]. In humans, several studies found that Nrg4 mRNA levels were significantly lower in the subcutaneous and visceral adipose tissues of individuals with impaired glucose tolerance and T2DM. Moreover, Nrg4 mRNA or circulating Nrg4 levels were negatively related to the presence of MetS components [6, 8]. Taken together, these previous observations suggest that plasma Nrg4 concentration might be a useful biomarker of obesity-related metabolic and cardiovascular disorders.

In this report, we describe the first clinical study in a Chinese nT2DM population. Consistent with previous findings [6], we observed that plasma Nrg4 levels were significantly lower in nT2DM patients with MetS than in nT2DM patients without MetS. Plasma Nrg4 levels progressively decreased as the number of components of MetS increased. Prevalence of MetS was significantly lower in subjects in the highest quartile of plasma Nrg4 concentration than in those in the lowest quartile. Plasma Nrg4 levels were positively correlated with a favorable lipid profile (high levels of HDL-C and apoA) and negatively correlated with an unfavorable metabolic profile (high levels of TG). Most importantly, we found that plasma Nrg4 concentration was independently associated with MetS, even after adjusting for all potential confounders. Collectively, these data demonstrate that Nrg4 is an independent protective factor for MetS and that decreased Nrg4 concentration may play an important role in the pathophysiology of MetS. However, the underlying mechanism remains unclear.

IR has been considered to be a key factor for development of MetS. HOMA-IR is a validated and widely used method to evaluate IR from FPG and FIns in large-scale and epidemiological studies [24, 25]. The TyG index was shown to be more sensitive and specific for IR than the euglycemic-hyperinsulinemic clamp [25]. The apoB/apoA ratio was significantly associated with IR in nondiabetic subjects in the United States, independent of traditional risk factors, MetS components, and inflammatory risk factors [26]. As expected, we found that compared to nT2DM patients without MetS, those with MetS had elevations of indicators of IR (FIns, HOMA-IR, apoB/apoA, and TyG index), suggesting that IR plays an important role in the development of MetS. Unfortunately, we did not find any difference in IR among patients in the four plasma Nrg4 quartiles, nor were plasma Nrg4 concentrations correlated with any index of IR. These data suggest that Nrg4 may not have a direct role in IR. Several previous studies demonstrated that Nrg4 overexpression could attenuate obesity-induced IR in animal models [7–9, 18]. In contrast, two recent clinical studies found that there were no differences in HOMA-IR among obese adults with different quartiles of serum Nrg4 concentration [9] and that circulating Nrg4 concentration was positively correlated with IR [19]. The discrepant findings between our study and those of the previous clinical trials might be due to methodological differences (e.g., in MetS definition, sample size, smoking habit, selected subgroups, and inclusion/exclusion criteria). In the present study, the OR for the association of plasma Nrg4 concentration with increased presence of MetS remained significant even after we adjusted for potential confounders, including all measures of IR. Hence, low plasma Nrg4 concentration seemed to add to the risk of MetS independently of IR, suggesting that plasma Nrg4 may protect against MetS via IR-independent mechanisms.

Accumulating experimental evidence suggests that all of the components of MetS, including IR, T2DM, hypertension, dyslipidemia, and visceral obesity, may increase oxidative stress and reduce antioxidant defenses [27]. Oxidative stress is thought to mediate the development of MetS [28]. GGT, a biomarker of hepatobiliary disease and alcohol consumption/abuse, is the principal enzyme responsible for extracellular catabolism of the antioxidant glutathione [29–31]. Elevated levels of GGT are involved in oxidative stress, lipid peroxidation, and mitochondrial dysfunction [31], and serum GGT activity changes in response to oxidative stress [29]. GGT levels were correlated positively with a marker of oxidative stress (F2-isoprostanes) and negatively with antioxidant levels [30]. Recent epidemiological and experimental data revealed that elevations in serum GGT concentration were associated with the presence of MetS and its components, even after adjustment for alcohol consumption and established risk factors [29, 31]. Consistent with these observations, we found that plasma GGT levels were lower in nT2DM patients with MetS than in those without MetS, suggesting that plasma GGT levels play an important role in the development of MetS. Surprisingly, we also found that plasma GGT levels were significantly different among patients in the four quartiles of plasma Nrg4 concentration, with nT2DM patients in the highest quartile exhibiting significantly lower levels of GGT than patients in the lowest quartile. Thus, there seems to be cross talk between plasma Nrg4 and GGT levels. Plasma Nrg4 levels remained significantly and negatively correlated with GGT after controlling for sex and age in a partial correlation analysis. Slattery et al. [32] recently reported that *Nrg4* gene expression was upregulated among patients with high oxidative balance scores (lower oxidative stress) compared to patients with lower scores. These results suggest that Nrg4 could potentially be used as a biomarker of oxidative stress and that decreased Nrg4 levels are likely a consequence of the augmented oxidative stress in MetS. Further study of this novel relationship between Nrg4 and GGT is warranted.

Chronic low-grade inflammation has been associated strongly with obesity and IR and, therefore, MetS [33]. A sensitive biomarker of low-grade systemic inflammation, hs-CRP is produced and released by the liver under the stimulation of proinflammatory cytokines [34]. Several studies support the concept that hs-CRP is a predictor of cardiovascular disease and T2DM [33, 35]. High concentrations of hs-CRP have been associated with MetS and its individual components in different populations [35]. WBC count is a routinely used marker of systemic inflammation that recently was reported to be associated with MetS and its individual components [36]. In nondiabetic individuals, lymphocyte count was associated with insulin sensitivity and adiposity [37, 38]. Neutrophil count correlates with hs-CRP concentration better than any other major white cell type [39]. Finally, numerous studies linked elevated levels of WBC subfractions (neutrophil and lymphocyte counts) and NLR to MetS [38]. We found that nT2DM patients with MetS had higher levels of several inflammatory markers (WBC, NLR, and hs-CRP) and lower lymphocyte counts than patients without MetS. These findings confirm the well-known association of these inflammatory markers with MetS in adults. In addition, nT2DM patients in the highest quartile of plasma Nrg4 levels had significantly lower WBC counts and hs-CRP levels than subjects in the lowest quartile. Thus, there seems to be a potential relationship between plasma Nrg4 levels and inflammation. Nrg4 may regulate the expression of proinflammatory cytokines in adipose tissue and influence circulating levels of WBCs and hs-CRP. Partial correlation analysis confirmed that the plasma Nrg4 levels were significantly and negatively correlated with hs-CRP level, WBC count, and neutrophil count after controlling for sex and age. Therefore, Nrg4 may play a key regulatory and anti-inflammatory role in development of MetS. Most importantly, hs-CRP and WBC were independently related to plasma Nrg4 levels. Taken together, these results demonstrate that proinflammatory cytokines may suppress Nrg4 expression in adipocytes. Thus, the reduction of plasma Nrg4 concentration is likely a consequence of augmented proinflammatory cytokine signaling in obesity [8]. Consistent with this hypothesis, recent studies demonstrated the interaction of Nrg4 with inflammation. For example, Bernard et al. [14] showed that Nrg4 expression was decreased in human inflammatory bowel diseases (Crohn disease and ulcerative colitis) and in mouse models of colitis, which may allow for macrophage persistence and ongoing inflammation. Similarly, Feng and Teitelbaum [40] reported reduced levels of Nrg4 in a mouse model of total parenteral nutrition-induced small intestinal inflammation. McElroy et al. [13] found that Nrg4 can protect against experimental necrotizing enterocolitis. Ma et al. [18] observed that Nrg4 overexpression reduced expression of genes encoding macrophage markers and the macrophage chemokine Mcp1 in liver and adipose tissues. Most recently, Schumacher et al. [15] suggested that Nrg4 expression in C57Bl/6 mice was suppressed by active inflammation during experimental colitis and rebounded during the recovery phase. Administration of exogenous Nrg4 reduced colonic macrophage numbers and ameliorated inflammation. Taken together, these previous and our present findings consistently raise the possibility that Nrg4 may have an important anti-inflammatory role through stimulating proinflammatory macrophage apoptosis and reducing gene expression of macrophage markers. The augmentation of inflammation due to Nrg4 downregulation may be important for the development of MetS. Future study is required to confirm our findings.

Obesity and dyslipidemia are the components of MetS that are most often seen in patients with T2DM. Obesity per se is an important risk factor of T2DM development and is associated with low-grade systemic inflammation and dyslipidemia. BMI is commonly used as a standard measurement of overall adiposity in adults [41]. Using both BMI and BF% provided more accurate measurements of the degree of obesity, especially for muscular athletes [42]. Elevated TG and reduced HDL-C levels reflect impaired lipid metabolism and are the main phenotypic features of dyslipidemia in diabetes [43]. Consistent with these findings, we found that compared to nT2DM patients without MetS, patients with MetS had higher levels of parameters of adiposity (BMI and BF%), TC, TG, and apoB, and lower levels of HDL-C and apoA, suggesting that obesity and dyslipidemia play key roles in development of MetS. In addition, patients in the highest quartile of Nrg4 concentration showed significantly lower BMIs and TG levels and significantly higher HDL-C and apoA levels than patients in the lowest quartile. Plasma Nrg4 levels were correlated negatively with TG and positively with HDL-C and apoA levels, even after adjusting for sex and age. These correlations reflect the role of Nrg4 in the modulation of weight and lipid metabolism. Our findings are inconsistent with those of two recent studies [19, 20], which showed that serum Nrg4 levels were or were not positively correlated with parameters of adiposity (BMI, WC, hip circumference, and neck circumference) and TG and were negatively correlated with HDL-C levels. The discrepant findings might be due to methodological differences. Meanwhile, Wang et al. [8] reported that Nrg4 mRNA expression was downregulated in the adipose tissues of several mouse models of obesity and inversely correlated with the percentage of body fat mass and liver fat content in humans. Nrg4 overexpression protected mice from diet-induced weight gain and adiposity and inhibited lipogenesis through liver X receptor (LXR) and sterol-regulatory element binding protein 1c (SREBP1c) and its target genes [8]. Similarly, Ma and colleagues [18] demonstrated that Nrg4 overexpression increased the expression of adipose triglyceride lipase (*Atgl*) gene and blocked hepatic lipid storage by inhibiting expression of peroxisome proliferator-activated receptor gamma (PPAR*γ*) and its target genes. Consistent with these findings, we found that plasma Nrg4 levels were significantly lower in patients with overweight or obesity or decreased HDL-C or elevated TG levels compared to their controls. Our multiple stepwise regression analyses showed that HDL-C level was independently correlated with plasma Nrg4 level. However, the physiological mechanisms underlying the relationship between Nrg4 and HDL-C remain obscure.

Our study has some limitations. Firstly, our findings must be interpreted with caution because the cross-sectional study design makes it hard to infer causality between plasma Nrg4 levels and MetS. Large prospective studies are needed to verify this potential causal relationship. Secondly, our findings may not be generalizable to other populations because the study was based on Chinese nT2DM patients at a single center. Further studies are warranted to determine the role of plasma Nrg4 in the development of MetS in other populations. Thirdly, the current gold standard to recognize IR, euglycemic-hyperinsulinemic clamp, is less frequently used and difficult to apply in large studies. As Nrg4 is thought to be related to IR, this study failed to examine the relationship between plasma Nrg4 levels and IR, as measured by HOMA-IR, apoB/apoA, and TyG index. Fourthly, our analyses were based on a single determination of plasma Nrg4 level, which is subject to random measurement error and may have underestimated the strength of the associations. Fifthly, newly diagnosed hypertension subjects were not excluded because of their small numbers. Their inclusion or exclusion would not have materially affected the results. Finally, BMI and BF% were used instead of WC to represent abdominal obesity. Although BMI might not represent fat accumulation accurately, BMI ≥ 25 kg/m^2^ was equivalent to WC = 85.5 cm (a value similar to the Japanese-specific cutoff point for abdominal obesity) as a predictor of the 5-year incidence of diabetes in Japanese–American men [44]. We believe that this CDS MetS criterion would not change the main implications of the present study.

Our study also has several strengths. This study included a relatively large number of patients. Furthermore, we tentatively defined MetS using CDS criteria appropriate for the Chinese population. Most importantly, our study is, to our knowledge, the first to evaluate the association between plasma Nrg4 level and MetS in Chinese nT2DM patients.

## 5. Conclusion

Our study examines a possible mechanistic association between the plasma Nrg4 level and MetS in nT2DM subjects. Plasma Nrg4 level could be a potential biomarker for the development of MetS. Further prospective studies are required to confirm the contribution of Nrg4 to the development of MetS. If this correlation were to be confirmed, increasing the levels of circulating Nrg4 might be crucial for the prevention or management of MetS and its related diseases.

## Figures and Tables

**Table 1:** Clinical and biochemical characteristics of nT2DM patients with and without MetS.

Covariate	Without MetS (*n* = 133)	With MetS (*n* = 178)	*P* value
Male/female	65/68	83/95	0.696
Age (years)	53.21 ± 10.36	54.06 ± 9.86	0.464
BMI (kg/m^2^)	22.71 ± 2.43	26.96 ± 3.24	<0.001
BF% (%)	26.10 ± 4.87	31.06 ± 4.61	<0.001
SBP (mmHg)	121.84 ± 9.90	129.79 ± 16.34	<0.001
DBP (mmHg)	71.99 ± 8.62	76.42 ± 9.48	<0.001
FPG (mmol/l)	10.11 ± 3.76	11.00 ± 3.59	0.036
2hPG (mmol/l)	17.38 ± 8.32	18.57 ± 5.64	0.017
HbA1c (%)	9.37 ± 2.83	9.38 ± 1.98	0.957
TC (mmol/l)	4.29 ± 0.93	4.77 ± 1.62	0.040
TG (mmol/l)	1.30 ± 0.62	2.85 ± 2.26	<0.001
HDL-C (mmol/l)	1.31 ± 0.29	1.00 ± 0.34	<0.001
LDL-C (mmol/l)	2.63 ± 0.80	2.78 ± 1.02	0.147
apoA (g/l)	1.53 ± 0.33	1.36 ± 0.26	<0.001
apoB (g/l)	0.84 ± 0.21	0.97 ± 0.29	<0.001
ALT (U/l)	22.03 ± 13.45	25.26 ± 13.87	0.006
AST (U/l)	21.72 ± 13.05	22.30 ± 10.53	0.139
GGT (U/l)	35.81 ± 6.21	40.75 ± 2.81	<0.001
ALP (U/l)	88.94 ± 44.47	83.06 ± 38.15	0.389
WBC (×10^9^/l)	6.52 ± 1.77	7.22 ± 2.56	0.004
Neutrophil (×10^9^/l)	4.45 ± 1.59	4.91 ± 2.42	0.148
Lymphocyte (×10^9^/l)	1.51 ± 0.55	1.68 ± 0.58	0.010
NLR	3.51 ± 0.25	3.58 ± 0.29	0.317
hs-CRP (mg/l)	5.98 ± 1.33	6.34 ± 0.96	0.007
FIns (*μ*U/ml)	8.45 ± 4.68	11.04 ± 7.26	0.002
HOMA-IR	3.80 ± 2.61	5.30 ± 3.92	<0.001
HOMA-IS	39.98 ± 37.33	39.63 ± 41.68	0.619
TyG index	4.94 ± 0.30	5.29 ± 0.47	<0.001
apoB/apoA	0.57 ± 0.19	0.73 ± 0.23	<0.001

BMI: body mass index; BF%: body fat percentage; SBP: systolic blood pressure; DBP: diastolic blood pressure; FPG: fasting plasma glucose; 2hPG: 2 h plasma glucose; HbA1c: glycated hemoglobin A1c; TC: total cholesterol; TG: triglyceride; HDL-C: high-density lipoprotein cholesterol; LDL-C: low-density lipoprotein cholesterol; apoA: apolipoprotein A; apoB: apolipoprotein B; ALT: alanine aminotransferase; AST: aspartate aminotransferase; GGT: gamma-glutamyltransferase; ALP: alkaline phosphatase; WBC: white blood cell; NLR: neutrophil to lymphocyte ratio; hs-CRP: high-sensitivity C-reactive protein; FIns: fasting plasma insulin; HOMA-IR: homeostasis model assessment of insulin resistance; HOMA-IS: homeostasis model assessment of *β*-cell insulin secretion; apoB/apoA: apolipoprotein A/apolipoprotein B; TyG: triglyceride glucose.

**Table 2:** Clinical and biochemical characteristics by quartile of plasma Nrg4 concentration in all nT2DM patients.

Covariate	Quartile 1	Quartile 2	Quartile 3	Quartile 4	*P* value
Sample size	77	78	79	77	
Nrg4 (ng/ml)	1.49 ± 0.31	2.30 ± 0.24^∗∗∗^	3.38 ± 0.38^∗∗∗^	5.99 ± 1.69^∗∗∗^	<0.001
Male/female	41/36	37/41	33/46	37/40	0.560
Age (years)	53.45 ± 9.61	52.56 ± 10.75	52.85 ± 10.52	55.95 ± 9.11	0.142
BMI (kg/m^2^)	26.09 ± 3.73	24.89 ± 3.70^∗^	24.98 ± 3.59	24.61 ± 3.23^∗^	0.054
BF% (%)	29.91 ± 5.39	28.61 ± 5.92	28.57 ± 5.11	28.68 ± 4.76	0.333
SBP (mmHg)	126.03 ± 15.36	127.37 ± 14.88	125.66 ± 14.56	126.52 ± 13.25	0.894
DBP (mmHg)	75.09 ± 10.89	75.09 ± 9.01	73.85 ± 8.87	74.08 ± 8.64	0.766
FPG (mmol/l)	10.48 ± 3.85	11.18 ± 3.95	10.38 ± 3.57	10.44 ± 3.37	0.496
2hPG (mmol/l)	18.29 ± 8.37	18.81 ± 7.40	17.41 ± 5.98	17.75 ± 5.69	0.605
HbA1c (%)	8.90 ± 2.24	9.67 ± 2.53	9.24 ± 2.13	9.61 ± 2.56	0.244
TC (mmol/l)	4.51 ± 1.37	4.43 ± 1.29	4.81 ± 1.76	4.52 ± 1.01	0.503
TG (mmol/l)	2.56 ± 2.48	2.47 ± 2.07	1.95 ± 1.49	1.76 ± 1.27^∗^	0.006
HDL-C (mmol/l)	1.02 ± 0.31	1.12 ± 0.38	1.17 ± 0.36^∗^	1.22 ± 0.34^∗∗^	0.005
LDL-C (mmol/l)	2.70 ± 0.86	2.50 ± 0.88	2.89 ± 1.08	1.22 ± 0.34	0.124
apoA (g/l)	1.36 ± 0.28	1.38 ± 0.25	1.48 ± 0.34	1.51 ± 0.31	0.020
apoB (g/l)	0.92 ± 0.26	0.91 ± 0.27	0.93 ± 0.31	0.91 ± 0.24	0.979
ALT (U/l)	24.50 ± 16.06	24.54 ± 13.07	24.35 ± 13.93	22.09 ± 11.77	0.551
AST (U/l)	23.26 ± 14.92	21.42 ± 8.91	22.93 ± 12.61	20.60 ± 9.16	0.555
GGT (U/l)	42.82 ± 9.55	45.13 ± 5.83	39.72 ± 4.55	26.80 ± 3.12^∗^	0.013
ALP (U/l)	86.55 ± 44.65	85.85 ± 47.29	88.42 ± 38.42	81.43 ± 32.54	0.563
WBC (×10^9^/l)	7.46 ± 2.17	7.19 ± 3.07	6.47 ± 1.74^∗∗^	6.59 ± 1.78^∗^	0.018
Neutrophil (×10^9^/l)	5.04 ± 1.96	5.03 ± 2.98	4.30 ± 1.60	4.50 ± 1.54	0.065
Lymphocyte (×10^9^/l)	1.69 ± 0.61	1.53 ± 0.57	1.65 ± 0.53	1.55 ± 0.57	0.240
NLR	3.81 ± 0.44	4.04 ± 0.57	3.06 ± 0.23	3.29 ± 0.20	0.096
hs-CRP (mg/l)	12.64 ± 1.98	6.68 ± 2.07^∗∗^	3.55 ± 0.88^∗∗∗^	1.94 ± 0.54^∗∗∗^	<0.001
FIns (*μ*U/ml)	9.75 ± 5.69	11.46 ± 7.94	8.89 ± 5.83	9.64 ± 5.74	0.175
HOMA-IR	4.44 ± 3.06	5.71 ± 4.54	4.15 ± 3.24	4.33 ± 2.68	0.113
HOMA-IS	42.64 ± 5.50	38.50 ± 3.89	34.47 ± 4.27	38.49 ± 4.32	0.755
TyG index	5.17 ± 0.47	5.21 ± 0.43	5.12 ± 0.48	5.05 ± 0.38	0.124
apoB/apoA	0.70 ± 0.24	0.68 ± 0.22	0.66 ± 0.24	0.63 ± 0.20	0.273
MetS, *n* (%)	55 (71.43)	46 (58.97)	43 (43.04)^∗∗^	34 (44.16)^∗∗^	0.007
Components of MetS					
Overweight or obesity, *n* (%)	43 (55.84)	35 (44.87)	40 (36.71)	29 (37.66)	0.130
Hypertension, *n* (%)	16 (20.78)	14 (17.95)	11 (13.92)	11 (14.29)	0.623
Elevated TG, *n* (%)	41 (53.25)	39 (50)	34 (34.18)	27 (35.06)	0.109
Decreased HDL-C, *n* (%)	31 (40.26)	24 (30.77)	20 (20.25)	16 (20.78)	0.050

^∗^ *P* < 0.05, ^∗∗^*P* < 0.01, ^∗∗∗^*P* < 0.001 versus quartile 1 group.

**Table 3:** Linear regression analysis of variables associated with plasma Nrg4 concentration in all nT2DM patients.

Variable	Simple Estimate	*P* value	Adjusted *P* value^∗^	Multiple Estimate	*P* value
Sex	0.040	0.487			
Age	0.099	0.081			
BF%	−0.049	0.392	0.272		
BMI	−0.127	0.025	0.263		
SBP	0.016	0.772	0.674		
DBP	−0.084	0.139	0.685		
TC	0.049	0.386	0.998		
TG	−0.208	<0.001	0.046		
HDL-C	0.194	0.001	0.016	0.119	0.035
LDL-C	0.030	0.600	0.785		
apoA	0.159	0.005	0.028		
apoB	−0.035	0.547	0.750		
apoB/apoA	−0.121	0.034	0.127		
ALT	−0.010	0.864	0.471		
AST	−0.023	0.688	0.534		
GGT	−0.119	0.037	0.049		
ALP	−0.052	0.360	0.333		
WBC	−0.156	0.006	0.012	−0.131	0.020
Neutrophil	−0.118	0.037	0.041		
Lymphocyte	−0.066	0.244	0.377		
NLR	−0.043	0.454	0.150		
hs-CRP	−0.447	<0.001	<0.001	−0.194	0.001
FPG	−0.005	0.935	0.857		
2hPG	−0.046	0.423	0.637		
HbA1c	0.043	0.449	0.103		
TyG index	−0.097	0.087	0.215		
FIns	−0.051	0.366	0.477		
HOMA-IR	−0.039	0.493	0.800		
HOMA-IS	−0.035	0.538	0.637		

^∗^Adjusted for sex and age.

**Table 4:** Odds ratios for the presence of MetS according to plasma Nrg4 concentration in all nT2DM patients.

	Increased MetS OR (95% CI)	*P* value
Model 1	0.85 (0.752–0.961)	0.009
Model 2	0.833 (0.717–0.967)	0.016
Model 3	0.750 (0.574–0.980)	0.035
Model 4	0.741 (0.555–0.990)	0.042
Model 5	0.683 (0.492–0.947)	0.022
Model 6	0.560 (0.374–0.837)	0.005

OR: odds ratio; CI: confidence interval. Model 1 unadjusted; model 2 adjusted for sex, age, and parameters of adiposity (BMI and BF%); model 3 adjusted for factors listed in model 2 plus parameters of glycolipid metabolism (SBP, DBP, FPG, 2hPG, HbA1c, TC, TG, HDL-C, LDL-C, apoA, and apoB); model 4 adjusted for factors listed in model 3 plus measures of insulin resistance (apoB/apoA, TyG index, FIns, HOMA-IR, and HOMA-IS); model 5 adjusted for factors listed in model 4 plus markers of inflammation (WBC count, neutrophil count, lymphocyte count, NLR, and hs-CRP); model 6 adjusted for factors listed in model 4 plus biomarkers of liver function (ALT, AST, GGT, and ALP).

## References

[1] Kim J.-Y., Ahn S. V., Yoon J.-H. (2013). Prospective study of serum adiponectin and incident metabolic syndrome: the ARIRANG study. *Diabetes Care*.

[2] Tao L. X., Li X., Zhu H. P. (2014). Association of hematological parameters with metabolic syndrome in Beijing adult population: a longitudinal study. *Endocrine*.

[3] Siu P. M., Yuen Q. S. (2014). Supplementary use of HbA1c as hyperglycemic criterion to detect metabolic syndrome. *Diabetology & Metabolic Syndrome*.

[4] Yang M., Liu R., Li S. (2013). Zinc-*α*2-glycoprotein is associated with insulin resistance in humans and is regulated by hyperglycemia, hyperinsulinemia, or liraglutide administration: cross-sectional and interventional studies in normal subjects, insulin-resistant subjects, and subjects with newly diagnosed diabetes. *Diabetes Care*.

[5] Dai Y. N., Zhu J. Z., Fang Z. Y. (2015). A case-control study: association between serum neuregulin 4 level and non-alcoholic fatty liver disease. *Metabolism*.

[6] Cai C., Lin M., Xu Y., Li X., Yang S., Zhang H. (2016). Association of circulating neuregulin 4 with metabolic syndrome in obese adults: a cross-sectional study. *BMC Medicine*.

[7] Pfeifer A. (2015). NRG4: an endocrine link between brown adipose tissue and liver. *Cell Metabolism*.

[8] Wang G. X., Zhao X. Y., Meng Z. X. (2014). The brown fat-enriched secreted factor Nrg4 preserves metabolic homeostasis through attenuation of hepatic lipogenesis. *Nature Medicine*.

[9] Jiang J., Lin M., Xu Y. (2016). Circulating neuregulin 4 levels are inversely associated with subclinical cardiovascular disease in obese adults. *Scientific Reports*.

[10] South J. C. M., Blackburn E., Brown I. R., Gullick W. J. (2013). The neuregulin system of ligands and their receptors in rat islets of Langerhans. *Endocrinology*.

[11] Christian M. (2014). Transcriptional fingerprinting of “browning” white fat identifies NRG4 as a novel adipokine. *Adipocytes*.

[12] Wang G. X., Zhao X. Y., Lin J. D. (2015). The brown fat secretome: metabolic functions beyond thermogenesis. *Trends in Endocrinology and Metabolism*.

[13] McElroy S. J., Castle S. L., Bernard J. K. (2014). The ErbB4 ligand neuregulin-4 protects against experimental necrotizing enterocolitis. *The American Journal of Pathology*.

[14] Bernard J. K., McCann S. P., Bhardwaj V., Washington M. K., Frey M. R. (2012). Neuregulin-4 is a survival factor for colon epithelial cells both in culture and in vivo. *The Journal of Biological Chemistry*.

[15] Schumacher M. A., Hedl M., Abraham C. (2017). ErbB4 signaling stimulates pro-inflammatory macrophage apoptosis and limits colonic inflammation. *Cell Death & Disease*.

[16] Villarroya F., Cereijo R., Villarroya J., Giralt M. (2017). Brown adipose tissue as a secretory organ. *Nature Reviews Endocrinology*.

[17] Rosell M., Kaforou M., Frontini A. (2014). Brown and white adipose tissues: intrinsic differences in gene expression and response to cold exposure in mice. *American Journal of Physiology-Endocrinology and Metabolism*.

[18] Ma Y., Gao M., Liu D. (2016). Preventing high fat diet-induced obesity and improving insulin sensitivity through neuregulin 4 gene transfer. *Scientific Reports*.

[19] Kang Y. E., Kim J. M., Choung S. (2016). Comparison of serum neuregulin 4 (Nrg4) levels in adults with newly diagnosed type 2 diabetes mellitus and controls without diabetes. *Diabetes Research and Clinical Practice*.

[20] Chen L.-L., Peng M.-M., Zhang J.-Y. (2016). Elevated circulating neuregulin4 level in patients with diabetes. *Diabetes/Metabolism Research and Reviews*.

[21] Metabolic syndrome study cooperation group of Chinese diabetes society (2004). Suggestions about metabolic syndrome of Chinese diabetes society. *Chinese Journal of Diabetes*.

[22] Yang W., Lu J., Weng J. (2010). Prevalence of diabetes among men and women in China. *The New England Journal of Medicine*.

[23] Deurenberg P., Weststrate J. A., Seidell J. C. (1991). Body mass index as a measure of body fatness: age- and sex-specific prediction formulas. *The British Journal of Nutrition*.

[24] Matthews D. R., Hosker J. P., Rudenski A. S., Naylor B. A., Treacher D. F., Turner R. C. (1985). Homeostasis model assessment: insulin resistance and beta-cell function from fasting plasma glucose and insulin concentrations in man. *Diabetologia*.

[25] Guerrero-Romero F., Simental-Mendía L. E., González-Ortiz M. (2010). The product of triglycerides and glucose, a simple measure of insulin sensitivity. Comparison with the euglycemic-hyperinsulinemic clamp. *The Journal of Clinical Endocrinology and Metabolism*.

[26] Sierra-Johnson J., Romero-Corral A., Somers V. K. (2007). ApoB/apoA-I ratio: an independent predictor of insulin resistance in US non-diabetic subjects. *European Heart Journal*.

[27] Chen S. J., Yen C. H., Huang Y. C., Lee B. J., Hsia S., Lin P. T. (2012). Relationships between inflammation, adiponectin, and oxidative stress in metabolic syndrome. *PLoS One*.

[28] West I. C. (2000). Radicals and oxidative stress in diabetes. *Diabetic Medicine*.

[29] Kawamoto R., Kohara K., Tabara Y., Miki T., Otsuka N. (2009). Serum gamma-glutamyl transferase levels are associated with metabolic syndrome in community-dwelling individuals. *Journal of Atherosclerosis and Thrombosis*.

[30] Lee D. S., Evans J. C., Robins S. J. (2007). Gamma glutamyl transferase and metabolic syndrome, cardiovascular disease, and mortality risk: the Framingham Heart Study. *Arteriosclerosis, Thrombosis, and Vascular Biology*.

[31] Wei D., Chen T., Li J. (2015). Association of serum gamma-glutamyl transferase and ferritin with the metabolic syndrome. *Journal of Diabetes Research*.

[32] Slattery M. L., Pellatt D. F., Mullany L. E., Wolff R. K. (2015). Differential gene expression in colon tissue associated with diet, lifestyle, and related oxidative stress. *PLoS One*.

[33] Devaraj S., Swarbrick M. M., Singh U., Adams-Huet B., Havel P. J., Jialal I. (2008). CRP and adiponectin and its oligomers in the metabolic syndrome: evaluation of new laboratory-based biomarkers. *American Journal of Clinical Pathology*.

[34] Lin C. C., Kardia S. L., Li C. I. (2010). The relationship of high sensitivity C-reactive protein to percent body fat mass, body mass index, waist-to-hip ratio, and waist circumference in a Taiwanese population. *BMC Public Health*.

[35] den Engelsen C., Koekkoek P. S., Gorter K. J., van den Donk M., Salome P. L., Rutten G. E. (2012). High-sensitivity C-reactive protein to detect metabolic syndrome in a centrally obese population: a cross-sectional analysis. *Cardiovascular Diabetology*.

[36] Babio N., Ibarrola-Jurado N., Bulló M. (2013). White blood cell counts as risk markers of developing metabolic syndrome and its components in the PREDIMED study. *PLoS One*.

[37] Wu H., Ghosh S., Perrard X. D. (2007). T-cell accumulation and regulated on activation, normal T cell expressed and secreted upregulation in adipose tissue in obesity. *Circulation*.

[38] Lorenzo C., Hanley A. J., Haffner S. M. (2014). Differential white cell count and incident type 2 diabetes: the insulin resistance atherosclerosis study. *Diabetologia*.

[39] Kaur H., Adams-Huet B., Smith G., Jialal I. (2013). Increased neutrophil count in nascent metabolic syndrome. *Metabolic Syndrome and Related Disorders*.

[40] Feng Y., Teitelbaum D. H. (2012). Epidermal growth factor/TNF-*α* transactivation modulates epithelial cell proliferation and apoptosis in a mouse model of parenteral nutrition. *American Journal of Physiology. Gastrointestinal and Liver Physiology*.

[41] Guo J., Zhang X., Wang L., Guo Y., Xie M. (2013). Prevalence of metabolic syndrome and its components among Chinese professional athletes of strength sports with different body weight categories. *PLoS One*.

[42] Chen C., Lu F. C., Department of Disease Control Ministry of Health, PR China (2004). The guidelines for prevention and control of overweight and obesity in Chinese adults. *Biomedical and Environmental Sciences*.

[43] Cui H. B., Wang S. H., Wang D. Q. (2007). Modified classic risk factors for coronary artery disease in Chinese Han population. *Chinese Medical Sciences Journal*.

[44] McNeely M. J., Boyko E. J., Shofer J. B., Newell-Morris L., Leonetti D. L., Fujimoto W. Y. (2001). Standard definitions of overweight and central adiposity for determining diabetes risk in Japanese Americans. *The American Journal of Clinical Nutrition*.

